# Embalming Match

**Published:** 1882-10-20

**Authors:** 


### EDITORIALS AND MISCELLANEOUS.

### *EMBALMING MATCH.*

It appears that the undertakers had a convention in New York, and among other things an embalming match has been appointed for that city to ascertain the best method of embalming or preserving the dead body.

